# MicroRNAs in Prostate Cancer: Implications for Treatment Response and Therapeutic Targets

**DOI:** 10.3390/cancers15205023

**Published:** 2023-10-17

**Authors:** Mohamed Ali Hussein, Gnanasekar Munirathinam

**Affiliations:** 1 Department of Pharmaceutical Services, Children’s Cancer Hospital Egypt 57357, Cairo 11562, Egypt; mohamedalihussien8554@gmail.com; 2 Department of Biology, School of Sciences and Engineering, American University in Cairo, New Cairo 11835, Egypt; 3 Department of Biomedical Sciences, College of Medicine, University of Illinois, Rockford, IL 61107, USA

## 1. Introduction

Cancer resistance to treatment is very common, represents one of the most significant challenges in the last few decades, and continues to impede all efforts to cure cancer. Resistance to treatment is a shared problem that arises in response to chemotherapy, radiotherapy, and targeted therapy in almost all types of cancer [1,2,3,4]. Despite recent advances in treatment strategies to combat cancer, resistance remains the principal cause of cancer-associated mortality, with approximately 90% of cases associated with drug resistance [5]. Resistance occurs when cancer cells evade the lethal effect of cancer therapy and results in disease progression and metastasis [6]. Resistance is a multifaceted problem that includes several factors that intricately interact to elicit the phenotypic changes associated with therapy resistance, such as tumor heterogeneity, the emergence of cancer stem cell subpopulations, drug efflux, molecular genetic alterations, epithelial–mesenchymal transition (EMT), the activation of oncogenic pathways, the epigenetic alteration associated with resistance, and tumor microenvironment [5,7,8,9,10]. Combating therapy resistance requires the usage of novel approaches for early diagnosis that help in stratifying patients based on their response to treatment and developing innovative therapeutic approaches that aim to reverse therapy resistance.

Prostate cancer (PCa) is the most common cancer occurring in men and the second leading cause of cancer mortality among men in the United States [11]. In the early stages, PCa is indolent and curable, while in advanced stages, the emergence of resistance is rampant and leads to treatment failure. Genetic and epigenetic alterations are frequently seen in PCa and significantly contribute to therapy resistance [12]. Doldi et al. [13] conducted a thorough review of the literature on the role of microRNAs as epigenetic determinants of treatment response and potential therapeutic targets in PCa. They shed light on the pivotal role of microRNAs (miRNAs), a group of noncoding RNAs that can regulate gene expression of several essential processes in PCa, including cell proliferation, cell cycle regulation, DNA damage response (DDR), autophagy, EMT, and metastasis, in modulating the treatment response [14]. The importance of this review is that it uncovers the common alteration associated with the development of resistance to anti-cancer treatment. In addition, it thoroughly examines the key miRNAs that are intricately involved in the development of resistance to anti-cancer therapy in PCa. In addition, the authors explore whether miRNAs can be used as novel biomarkers for the diagnosis of chemoresistance or innovative targets that can be used to reverse chemoresistance in PCa. 

The authors, in their review article, started by giving an overview of the treatment options commonly used in different stages of PCa and the effect of molecular alteration that impacts the evolution of resistance. Surgical castration via radical prostatectomy and radiation therapy (RT), such as intensity-modulated external beam radiation therapy and image-guided radiation therapy, both represent the gold standard of RT; conformal radiotherapy (3D-CRT) and brachytherapy can be used alone in low- and intermediate-risk patients or as adjuvant therapy with radical prostatectomy in locally advanced PCa. RT works by creating DNA double-strand breaks and generating reactive oxygen species (ROS) [15]. Nevertheless, resistance to RT can occur due to various molecular alterations, such as alteration in the DDR elements, like p53/MDM2, ATM/ATR, and PARP-1, or alteration in the apoptosis pathways, like Bax and Bcl-2. In addition, alterations in tissue hypoxic status, particularly changes in the hypoxia-inducible factor HIF1α and its related pathways (including the PI3K/Akt/mTOR pathway, the Wnt/β-catenin pathway, NADPH oxidase (NOX), and the Hedgehog pathway), have significantly impacted the development of resistance to RT. Moreover, alterations in EMT are characterized by an upregulation of EMT proteins, like vimentin, ZEB-1, and snail. This alteration is also associated with various pathways linked to EMT, including TGF-β, Wnt, EGFR/PI3K/Akt, Hedgehog, Notch, MAPK, and p21-PAK1, which have a significant impact on the emergence of resistance to RT [16]. 

Next in their review, the authors discussed chemical castration using androgen deprivation therapy (ADT), including luteinizing hormone-releasing hormone agonists and antagonists and antiandrogen therapy that castrates the level of androgen. Nonetheless, resistance often emerges after 2–3 years of treatment, leading to the development of a lethal status of the disease known as castration-resistant prostate cancer (CRPC) [17]. The resistance is often linked to changes in the androgen receptor (AR) caused by mutations or increased expression of AR splice variants, as well as changes in associated molecules that can activate the pathways capable of bypassing AR, such as upregulation in NF-κB, IGF-1, and EGR, and PTEN loss. Furthermore, the authors explored the use of chemical agents in fighting PCa, specifically docetaxel and cabazitaxel, as well as the immunotherapeutic agents Sipuleucel-T and Radium-233 for the treatment of metastatic castration-resistant prostate cancer (mCRPC). Resistance to docetaxel is frequent and can be linked to alterations in ATP binding cassette (ABC) transporters, β-tubulin, and EMT [18].

The authors further delved into miRNAs and their crucial role in cancer and treatment response. The miRNAs are a type of non-coding RNA composed of 18–25 nucleotides and can regulate gene expression for various vital pathways by binding with mRNA [19]. This binding can lead to the complete decay of the mRNA, causing a total silence of the target gene or partial inhibition, which results in the blocking of protein translation and a decrease in the protein level of the target gene. They can either work by lowering the expression of tumor suppressor genes and promoting cancer, known as oncomiRs, or by inhibiting oncogenes and, thus, preventing cancer [20]. Taking this into consideration, miRNAs can be used as a novel target to combat cancer as well as valuable diagnostic markers. A plethora of evidence revealed the dysregulation of miRNAs in PCa by comparing the expression level of normal and tumor tissues. Certain miRNAs can work as a tumor suppressor by interfering with cell proliferation, migration, and promoting cell death. Of note, miR-15 and miR-16 were the first microRNAs reported in PCa. They inhibit cancer cell proliferation and invasion by interfering with the cyclin D1 and WNT3A signaling pathways. Also, miR-205 is another crucial miRNA due to its ability to counteract the effect of cancer-associated fibroblasts and inhibit EMT in PCa cells. Likewise, it plays a pivotal role in preserving the basement membrane, which further enhances its ability to inhibit EMT. Moreover, miR-34a is frequently downregulated in PCa due to its pro-apoptotic role, making it a prime target for human miRNA therapy. Although miR-21 is known to be an oncomiR that can be a promising target in the treatment of PCa, its role in PCa carcinogenesis is still a subject of debate. The authors highlighted the inconsistencies in its role, and further research is warranted to fully exploit it in PCa [13].

The authors also surveyed the role of miRNAs in regulating the response to RT. Although there are inconsistencies in the reports of ionizing radiation (IR) affecting miRNA expression in vitro, miRNAs can either intensify or mitigate the cell response to RT by interfering with essential processes in PCa cells. One of these processes is DDR. Several miRNAs can make PCa more sensitive to RT by interfering with various DDR genes, such as miR-890 and miR-744-3p, inhibiting homologous recombination, like miR-99a, miR-100, and miR-145, interfering with PKCέ and EGFR, and interrupting non-homologous end-joining (NHEJ) initiation like miR-205 or inhibiting DNA repair proteins such as miR-521. Moreover, miRNAs can also regulate cell response to RT through cell cycle checkpoints, including cyclins, cyclin-dependent kinases, cyclin-dependent kinase inhibitors, and transcription factors. Interestingly, miR-16-5p and miR-107 has been shown to sensitize PCa cells for RT by targeting cyclin D1 and pleiotropic growth factor GRN, respectively. Likewise, miR-449 sensitizes cells to RT via targeting pRb/E2F1 and c-Myc in vitro and in vivo. On the other hand, miR-16-5p and miR-95 mediate the resistance of PCa to RT by increasing p21 and inhibiting SGPP1, respectively [13]. 

In addition, the authors discussed how miRNAs regulate principal cell death pathways, including apoptosis and autophagy, in the PCa cell response to RT. In regard to apoptosis, miR-541-3p sensitizes the PCa cell RT and induces PCa cell apoptosis via targeting HSP27, while miR-498 inhibits apoptosis and induces radioresistance via modulating PTEN. On the other hand, autophagy modulates cell survival and enhances resistance to RT. Specifically, miR-32 promotes cell survival and induces autophagy by regulating DAB2IP, therefore inducing radioresistance. In contrast, miR-124 and miR-144 inhibit apoptosis and sensitize PCa cells to RT via targeting PIM1 kinase. Likewise, miR-205 and miR-30a also induce radiosensitivity by inhibiting autophagy through mediating TP53INP1. Notably, miR-205 and miR-875-5p suppress EMT by targeting ZEB1 and PKCέ, respectively. PKCέ impairs the NHEJ pathway via EGFR. In addition, miR-1272 induces radiosensitivity via indirect targeting of EGFR [13]. 

The authors further proceeded to discuss the implications of miRNAs in drug response to ADT and chemotherapy. Importantly, miRNAs play a crucial role in modulating several pathways related to drug response and resistance to ADT and chemotherapy. This includes reactivation of the AR signaling or hijacking other pathways to bypass the AR signal. miR-185 is one of several miRNAs that is downregulated in PCa tumor samples compared to control and can overcome androgen dependency in PCa cells through directly binding to the 3′UTR of AR mRNA or inhibiting the AR co-activator bromodomain-containing 8 isoform 2, and sterol regulatory element-binding protein-1 (SREBP-1). The miR-221/222 cluster has a controversial role in PCa cells, which depends on AR status, as suggested by several studies that investigated the expression of miR-221/222 in both AR-independent PCa cell models, such as PC-3 and DU145, and AR-sensitive LNCaP, as well as in vivo mouse models. Moreover, the miR-30 family of miRNAs can directly bind to the 3'UTR of AR or its splice variant ARv7 and interfere with their transcripts. The inhibition of several members of this family in an androgen-deprived environment enhances PCa cell proliferation. In addition, miR-34c, miR-449b, and miR-124 directly interfere with the expression of ARv7a and ARv4 transcripts through regulation of the splice variant process [13].

Furthermore, the authors discussed how miRNAs affect drug response by overriding the cell cycle arrest checkpoints and escaping apoptosis. In this regard, miR-143 has been linked to evading apoptosis by interfering with ERK5. Its ectopic expression was associated with increased sensitivity to docetaxel via targeting the KRAS pathway. In addition, several miRNAs, such as miR-223-3p, miR-323, miR-375, and miR-148a, were linked to taxane resistance by interfering with apoptosis. Moreover, miR-21 is often overexpressed in cancer and renders PCa resistant to docetaxel by interfering with proapoptotic proteins and downstream pathways. Likewise, miR-34a sensitizes PCa cells to paclitaxel treatment by targeting the antiapoptotic proteins SIRT1 and Bcl-2. Similarly, miR-205 can sensitize PCa cells to cisplatin and doxorubicin by interfering with the antiapoptotic protein Bcl-2 [13]. 

The role of EMT in chemoresistance is well established in cancer [21]. Several miRNAs can interfere with the EMT process and impact drug response in PCa. One of those is the miR-200 superfamily, especially miR-200c, which can render PCa sensitive to docetaxel by interfering with the EMT process by downregulating the mesenchymal markers ZEB1 and ZEB2 while upregulating the expression of the epithelial marker E-cadherin. Similarly, another member, miR-200b, enhances PCa sensitivity by promoting the expression of Bim-1 and subsequently induces apoptosis. In addition, miR-128 overexpression sensitizes PCa cells to cisplatin via the inactivation of ZEB1 [13]. 

Furthermore, the authors discussed the role of miRNAs in regulating drug efflux and, subsequently, implicating drug response. The involvement of miRNAs in regulating ABC transporters includes intricate interaction with long non-coding RNAs (lncRNA) as well as target genes. In PCa, the lncRNA NEAT1 sponges miR-204 and miR-34a, resulting in the activation of ACSL4 and downstream ABCG2 and ABCC4 transporters, leading to the development of resistance to docetaxel. Additionally, another lncRNA, DANCER, induces resistance to docetaxel by sponging miR-34a, which results in the activation of JAG1 and, subsequently, the upregulation of ABCB1 and ABCC4 transporters [13]. 

Finally, the authors delve into exploring the role of miRNAs in the development of neuroendocrine prostate cancer (NEPC). In this context, several miRNAs, including miR-663, miR-708, and miR-375, are associated with the development of NEPC by upregulating neuroendocrine genes. Additionally, the miR-106a~363 cluster is responsible for driving NEPC by regulating various factors essential for NEPC development, such as Aurora Kinase A, E2F1, N-Myc, and STAT3 [13].

## 2. Conclusions

The evidence presented in this review highlights the pivotal role of miRNAs in the development and progression of PCa. It can also impact the cell response to RT, ADT, and chemotherapy. Modulating miRNA expression with inhibitors or mimics can hinder tumor growth and improve treatment response, especially in aggressive PCa subtypes, such as CRPC and NEPC. These make them useful as reliable diagnostic and prognostic markers. However, several challenges hinder the translation into human cancer therapy. One major problem is the lack of a safe and efficient delivery system in addition to identifying the intricate interaction between miRNA and hub genes involved in PCa development and progression. Moreover, the safety profile of miRNA-based molecules needs to be evaluated, and more studies are warranted to study the pharmacokinetics of miRNA inhibitors and mimics and their effect on human health and disease status.
